# Endemic Plant Species Conservation: Biotechnological Approaches

**DOI:** 10.3390/plants9030345

**Published:** 2020-03-09

**Authors:** Natacha Coelho, Sandra Gonçalves, Anabela Romano

**Affiliations:** MED—Mediterranean Institute for Agriculture, Environment and Development, University of Algarve, Faculty of Sciences and Technology, Campus de Gambelas, Ed. 8, 8005-139 Faro, Portugal; nrcoelho@ualg.pt

**Keywords:** cryopreservation techniques, explant, *ex situ* conservation, genetic stability, micropropagation, shoot tips, seeds, vitrification

## Abstract

Endemic plant species are usually more vulnerable to anthropogenic threats and natural changes and, therefore, hold a higher extinction risk. The preservation of these species is a major concern on a worldwide context and *in situ* protection alone will not guarantee their conservation. *Ex situ* conservation measures must be undertaken to support the conservation of these species, and seed banking is the more efficient and cost-effective method. However, when seed banking is not an option, alternative approaches should be considered. Biotechnological tools provide new and complementary options for plant conservation including short-, medium-, and long-term strategies, and their application for plant species conservation has increased considerably in the last years. This review provides information about the status of the use biotechnology-based techniques for the conservation of endemic plant species. Particular attention is given to cryopreservation, since is the only long-term *ex situ* conservation strategy that can complement and support the other conservation measures. The cryopreservation of plant genetic resources is, however, more focused on crop or economically important species and few studies are available for endemic plant species. The plant material used, the cryopreservation methods employed, and the assessment of cryogenic effects are reviewed. The reasons to explain the difficulties in cryopreserving these species are discussed and new strategies are proposed to facilitate and increase the interest on this matter. We expect that further studies on the conservation of endemic plant species will increase in a near future, thus contributing to maintain these valuable genetic resources.

## 1. The Conservation of Endemic Plant Species

Plants are vital for life on earth and a crucial element in all ecosystems. Despite their importance, all over the world, plant biodiversity is at risk and every year the number of threatened species increases dramatically [1]. The loss of natural populations or even entire species is usually related to the destruction and alteration of their habitats, as a consequence of human overexploitation, and more recently, pollution, and climate changes, leading to the loss of genetic diversity [2,3,4]. Many of these species are endemic, therefore unique, and often only a few and small wild populations resist [4,5].

An endemic species can be defined as a species that occurs naturally and exclusively, and it is highly adapted to a specific geographic area [6,7,8,9]. According to the size and limits of that area, these species can be classified as “local endemic” (restricted to a small area), “provincial endemic” (restricted to the limits of a province), “national endemic” (restricted to the limits of a nation), “regional endemic” (restricted to a geographical region) and “continental endemic” (restricted to a continent) [9,10]. A set of characteristics, alone or together, can be found in most endemic species, which make them more vulnerable than others to anthropogenic threats and/or natural changes: restricted distribution, one or few populations, small population size, declining population size, excessive collection by humans, short reproduction capacity, specific habitat conditions, and necessity of stable and constant environments. The more of these characteristics these species display, the more vulnerable they are to extinction [9]. Hence, endemic species should be carefully monitored and managed, and their conservation considered a global priority [8,9].

The most important source of information concerning the global conservation status of species is the IUCN (International Union for Conservation of Nature) Red List of Threatened Species [9,11], and according to the latest numbers, there are 15,774 threatened plant species, out of the 38,630 species evaluated so far, of the 422,683 described species [11]. Many plant species (more than 75%) are endemic to the 35 Global Biodiversity Hotspots and conservation efforts pointed to these regions could greatly contribute to reduce the loss of these unique species [12,13].

*In situ* conservation, the conservation of ecosystems and biodiversity in their natural habitats, is the most appropriate conservation approach for the preservation of species, including endemic species, because it preserves the original genetic and geographical centers of biodiversity [14,15]. However, for a more complete and effective conservation program, different strategies and methods should be implemented to complement and support *in situ* protection [15]. In fact, *ex situ* conservation, the conservation of biodiversity outside its natural habitats, is sometimes the only option for the preservation of rare and endemic species [4,5,14]. Conventional seed bank storage (dry storage at −20 °C) is the simplest and most efficient method for *ex situ* conservation of plant germplasm, and it is also the best choice because it preserves genetically diverse material [16]. However, not all plant species can be preserved by this technique. Species with recalcitrant (desiccation-sensitive) and intermediate (relatively desiccation-tolerant) seeds cannot stand the desiccation conditions and cold storage without losing viability; only orthodox seeds (desiccation-tolerant) are able to do so [14]. Besides, there are plant species that do not produce seeds and are propagated vegetatively. Furthermore, there are others that, even though they can produce seeds, are highly heterozygous. For these plant species, the traditional *ex situ* conservation method is in the form of field collections [14,17]. But this method also has its limitations: it usually requires large areas of land, is labor intensive, and collections are unprotected from plagues and natural disasters [17]. In addition, field collections are mainly used to preserve crop species. Thus, it is important to implement alternative approaches for the preservation of these species, such as biotechnological methods. Even so, the use of alternative conservation methods should not only be considered for vegetatively propagated and non-orthodox seeded species. In some cases, in addition to traditional conservation methods, it is necessary to implement complementary strategies to augment the chances of survival even for species with orthodox seeds that are easily preserved in seed banks.

## 2. Biotechnological Approaches

**Figure 1:**
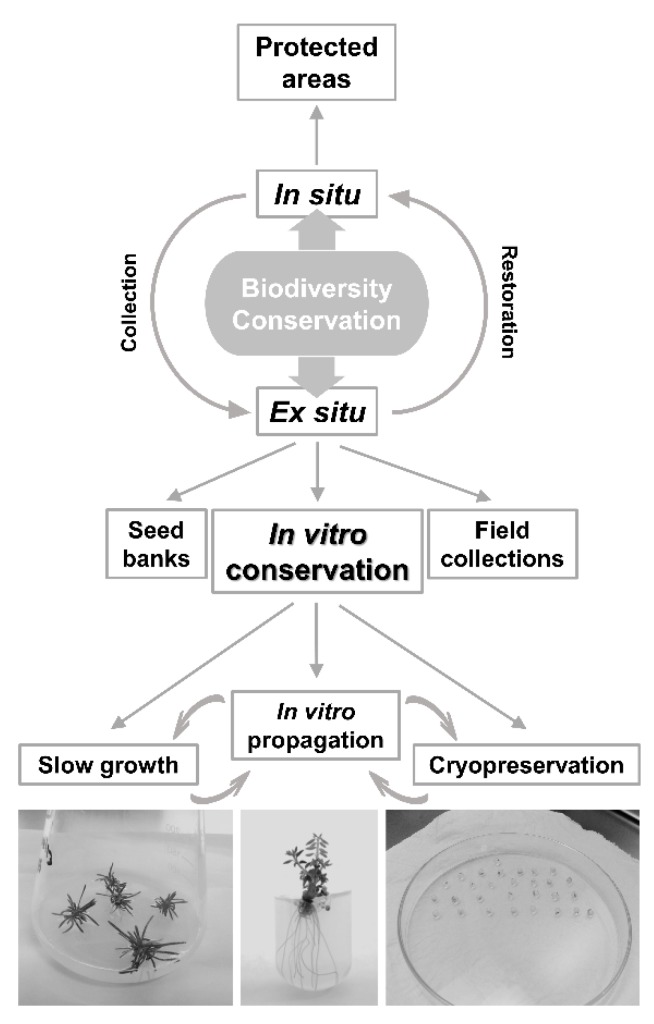
Schematic representation of different conservation strategies with focus on biotechnological-based techniques (adapted from [18]).

Biotechnology has given a major contribution to plant conservation and the purpose is not to replace the traditional conservation methods but to complement and improve the methods available (Figure 1) [14,18]. Plant conservation biotechnology comprises not only the conservation of plant genetic resources but also its management, characterization, and application (sustainable use) [18]. In particular, *in vitro* conservation, which is the maintenance of plant germplasm in culture collections using tissue culture technologies, can provide easy access for the evaluation, utilization, and safe exchange of plant material [4,14,15]. According to the storage duration, plant germplasm is maintained in culture collections for short-term periods, using standard tissue culture, or medium-term periods (slow growth), achieved by reducing growth. The long-term storage refers to the cryopreservation of plant material in liquid nitrogen (LN) [14]. There are a few reviews that discuss the use of biotechnological tools for the conservation of rare and endangered species, either presenting a synopsis on the subject [4,5,14] or focusing on a certain region/country [19,20] or groups of plants [21].

Nonetheless, and considering the significant progress on the use of biotechnological tools and their importance to complement other *ex situ* methods, the purpose of this review is to make a global overview of the conservation of endemic plant species with small populations and limited distribution based on *in vitro* culture techniques. Cryopreservation will be discussed in more detail, since it is the only technique that allows long-term conservation and, as far as our literature survey can ascertain, there is no comprehensive review on its application for the conservation of narrow endemic plant species, hereafter referred to just as endemic plant species.

## 3. Short- (*In Vitro* Propagation) and Medium-Term Preservation (Slow Growth)

*In vitro* propagation is considered an effective alternative to the conventional propagation methods, since it allows the recovery of numerous plants in a short period of time and limited space and facilitates the transportation of propagation material [14]. This is particularly useful in the case of rare and endangered species and plants that are difficult to propagate by conventional techniques or with slow propagation rates. Other advantage of this technique is the necessity of small amounts of starting plant material from the mother plant, which allows the mass propagation with scarce impact on wild habitats [14]. Reinforcement of wild plant populations using individuals raised *ex situ* is considered a valid means of reducing the risk of extinction of threatened species or populations [22]. Thus, the development of effective propagation protocols is fundamental in conservation programs. *In vitro* culture in normal growth conditions allows the short-term (active state growth) conservation of genetic material of rare and endangered plants being a fundamental step for the propagation of plants directly but also for the application of medium- (slow growth) and long-term conservation techniques (cryopreservation).

*In vitro* propagation is described for the propagation and conservation of many endemic plant species and some selected examples are presented in Table 1. Meristem culture and the differentiation of adventitious buds (organogenesis) are the methods usually used, although the first has more examples. Meristem culture is more adequate from the conservation point of view because meristematic cells are less differentiated and genetically more stable, originating genetic integral plants [23]. Many factors affecting the process, such as the basal medium composition, the plant growth regulators, the environmental parameters, as well as the explant type, have been investigated in order to optimize it for each species. A recent example of the development and optimization of an *in vitro* propagation protocol using seedlings as the initial explant was performed for *Eryngium viviparum* Gay, a small, biennial, and aquatic plant endemic to the Southwest Europe (namely France, Spain, and Portugal) [24]. The effect of two types of cytokinins (6- benzylaminopurine and kinetin) and different concentrations were tested to assess shoot multiplication. For shoot elongation and rooting, the authors tested different concentrations of sucrose and MS (Murashige and Skoog) [25] macronutrients. With the optimized protocol high rates for shoot multiplication, 5.1-5.8 new shoots, 100% rooting, and 96% acclimatization were obtained [24].

The maintenance of *in vitro* cultures in active growth (short-term conservation), involving the transference of cultures to fresh medium at regular and short periods (some weeks) is a laborious and expensive task. Slow growth storage is a strategy for the medium-term conservation of *in vitro* plant germplasm with the objective of reducing plant growth and, therefore, the number of sub-cultures required without affecting the viability of the explants. Using this strategy, cultures can be stored in the same medium for several months without subculture. Growth reduction is obtained by inducing osmotic stress, using growth retardants, reducing partial pressure of oxygen, or by changing the media composition (e.g., growth regulators, minerals) and/or the environmental conditions (e.g., reduction of temperature and/or light) [53]. Though the use of this technique in plant germplasm conservation significantly reduces costs and labor time, by reducing the number of subcultures, when compared to standard *in vitro* propagation, it still requires high storage space and some level of maintenance and the risk of somaclonal variation persists. There are several reports on the application of slow growth as a medium-term conservation strategy, but those are mainly focused on economic important crops. Regarding endemic plant species the studies are scarce [54,55], probably because efforts and resources have been concentrated in other strategies, like cryopreservation, that will be addressed in detail in the next section of this review.

## 4. Cryopreservation

Cryopreservation is a type of long-term *ex situ* conservation in which viable biological resources are stored at ultra-low temperatures, such as those of LN (–196 °C) and/or its vapor phase (–150 °C) [14,56,57]. Different types of plant cells, tissues, and organs can be cryopreserved, theoretically, without loss of their viability, because at these extreme low temperatures, metabolic processes cease, avoiding alterations and/or degeneration of plant material [14,57]. In addition, the material is stored in a small space, protected from contamination, and requires limited maintenance at a low cost. The cryopreservation techniques can be applied to different genotypes and the need for regular subcultures is eliminated, keeping the genetic stability of plant material [14,58,59].

To accomplish an efficient cryopreservation protocol, it is essential that plant cells can be cooled and recovered from ultra-low temperatures without injuries. The main cause of cellular damage is the transition of water into ice, producing ice crystals, during the cooling process [60]. Most plant cells/structures are extremely vulnerable to intracellular freezing, because of their cellular constitution and high-water content [14,57]. To prevent the formation of ice crystals, cells must be adequately dehydrated and exposed to cryoprotectant solutions, before cooling in LN. The exceptions are, for instance, orthodox seeds and dormant buds, that withstand desiccation better, and therefore can be directly cryopreserved without pretreatment [14].

The capacity to survive cryopreservation differs from species to species and even between different clonal lines of the same species. Protocols must be optimized individually according to the species/clone and explant typeto be cryopreserved to obtain the best recovery rates after cryostorage [20,61]. There are many cryopreservation studies concerning crops or economically relevant plant species, due to their value and the large knowledge of their growing conditions [14,62]. In the case of most endemic and wild species, they are highly variable and the information about their biology is scarce, which makes the process of developing a cryopreservation protocol more complex. Several factors/parameters that highly influence cryopreservation success are addressed below.

### 4.1. Explant Type

Different types of plant material, such as seeds, pollen, spores, cell suspensions, calli, shoot tips, somatic and zygotic embryos and dormant buds, can be cryopreserved. As shown in Table 2 and Table 3, most cryopreservation approaches developed for endemic plant species use seeds or shoot tips as the explant.

Though conventional seed storage is widely used for the conservation of orthodox seeds, cryopreservation presents as an alternative for the long-term conservation of seeds, which can prolong their longevity when compared to other storage temperatures. Several seeds from endemic plant species have been successfully cryopreserved by direct immersion into LN (Table 2). Considering the cryopreservation process used (dehydration followed by direct plunging into LN) these seeds can be described as orthodox. In most studies, there was no loss in seed viability and no significant differences were observed on the germination rate between cryopreserved and non-cryopreserved seeds [63,64,65,66].

On the contrary, the cryopreservation of recalcitrant and intermediate seeds, is a more complex procedure dependent on the seeds’ capacity to survive desiccation and cryogenic conditions. Usually these seeds are larger in size and instead of the complete seed, desiccated embryos or embryonic axes excised from the seed are often used for cryopreservation [72,73]. This approach was used to cryopreserve the germplasm of Nothapodytes nimmoniana (Graham) Melbbery, an endangered forest tree endemic to Western Ghats, India, with large and intermediate type seeds [74]. The embryonic axes of this species were dehydrated for 120 min and then immersed in LN, achieving a germination percentage of 60%. Pollen is another alternative for the conservation of species with recalcitrant or intermediate seeds. Pollinia of Luisia macrantha Blatt. & McCann, an endemic and endangered orchid of Western Ghats, India, was effectively cryopreserved with germination percentages around 55% [75].

For clonally propagated species, cryopreservation in seed form is not a valid option to preserve their germplasm. Besides, many endemic endangered species have small populations and/or scarce seed production, and the collection of seeds may compromise the species’ survival [76]. To cryopreserve these species, different explant types, other than seeds, have to be used, and shoot tips excised from *in vitro*-grown plants are a frequent option [17]. In fact, shoot tips are also the explant of choice for many other species. As previously mentioned, among the cryopreservation protocols developed for endemic species, the majority (not considering seed cryopreservation) uses shoot tips as plant material (Table 3). Shoot tips, containing the apical meristem, from where growth emerges, are organized and genetically stable structures. Cells within the apical meristem are slight differentiated, small and unvacuolated, and can resist desiccation and freezing better than highly vacuolated and differentiated cells [77,78]. Therefore, after cryostorage and rewarming, these characteristics enable direct shoot formation from cryopreserved shoot tips and maintenance of the genetic integrity [14,77,78]. Several endemic plant species, such as *Paraisometrum mileense* W. T. Wang (endemic to Yunan, China) [79], *Thymus moroderi* Pau ex Martínez (endemic to South-eastern Spain) [80,81], and *Tuberaria major* (Willk.) P. Silva and Rozeira (endemic to the south of Portugal) [82], were successfully cryopreserved using shoot tips, with survival (swollen green shoots or callus formation) or regrowth (normal shoot development) percentages above 50%.

Callus is another structure that can be used in cryopreservation. Despite being an unorganized tissue and more prone to genetic abnormalities, when compared to shoot tips, calli are easier and faster to handle, and the final amount of regenerated material can be higher [77,78]. Cryopreservation of shoot tips of *Loxocarya cinerea* R.Br., an endemic species to the southwest Western Australia, allowed a very low regeneration rate post-cryopreservation (below 5%) [78]. On the other hand, applying the same protocol to callus tissues, authors obtained survival percentages above 90%. There are other studies using diverse plant material for the cryopreservation of germplasm from endemic plant species. For instance, axillary buds and shoot tips were used as explants for the cryopreservation of *Hypericum richeri* ssp. *transsilvanicum* (Čelak) Ciocârlan, a plant species endemic to Transylvania, Romania, and the highest recovery percentage, 68%, was obtained using axillary buds [38]. Nodal segment was the explant chosen for the cryopreservation of *Lithodora rosmarinifolia* (Ten.) I. M. Johnst., a shrub endemic to Sicily, Italy [88]; *Plantago algarbiensis* Samp., endemic to the south of Portugal [70]; *Centaurium rigualii* Esteve, endemic to the southeast of the Iberian Peninsula [93]; and *Antirrhinum microphyllum* Rothm., endemic to Spain [92].

The cryopreservation of spores is recurrently a valid option for the long-term preservation of ferns. As with seeds, the conservation of spores allows the storage of a wider variety of genetic material. Besides, at sub-freezing temperatures, spores can be effectively stored as seeds [21]. Spores of *Pleopeltis lepidopteris* Langsd. & Fisch., a fern endemic to the south of Brazil, were successfully stored in LN, achieving germination percentages above 89% [96]. *Pteris adscensionis* Swartz, an endemic fern from Ascension Island, was chosen as a model species for the development of a standard cryopreservation procedure to be applied to other endemic and rare fern species from biodiversity hotspots and small islands over the world [21]. After germination of *P. adscensionis* spores, the obtained gametophytes were multiplied *in vitro* and further used for the cryopreservation trials. A survival of 48% was obtained for *P. adscensionis* after cryopreservation. When applying the same protocol to other fern species, the results were variable: 90, 86% and 47% of gametophytes survived for *Ctenitis pauciflora* (Kaulf.) Holttum. (not endemic), *Lepisorus longifolius* (Blume) Holttum. (not endemic) and *Macroglossum smithii* (Racib.) Campbell (endemic to Borneo), respectively, while no growth was achieved for *Marattia purpurescens* de Vriese (endemic to St. Helena). The explant type chosen to cryostorage germplasm from endemic plant species is highly dependent on the species and can have a major influence on the success of the cryopreservation process.

### 4.2. Cryopreservation Techniques

The classical cryopreservation techniques (controlled rate cooling, slow cooling, or two step cooling) are based on the use of cryoprotectants, to regulate cell water content, combined with slow cooling using a programmable freezer, to induce cell dehydration, followed by immersion in LN [14,61,97]. However, these techniques have been supplanted along the years for simpler and faster procedures and the most used techniques nowadays are vitrification and encapsulation-dehydration, and their derivates [14,56,57]. These techniques are based in the physical process named “vitrification” in which liquids solidify without crystallizing. There are several pre- and post-cryopreservation conditions/factors that can be considered to improve the efficiency of the cryopreservation process, namely the use of preconditioning strategies, standardization of the culture conditions of the *in vitro*-grown material (e.g., age, morphogenetic status, subculture period, light and nutritional requirements), and size of the explants, composition of the recovery medium, among others [97,98].

#### 4.2.1. Vitrification, Encapsulation-Dehydration, and Encapsulation-Vitrification

Vitrification and encapsulation-dehydration are conventional cryopreservation techniques successfully used in the cryopreservation of a wide range of plant species. In the vitrification technique plant material is exposed to highly concentrated cryoprotectant solutions for short periods. The most commonly used vitrification solutions are the glycerol-based ones developed by Sakai and co-workers [99] in a series known as plant vitrification solution (PVS) [100,101]. A complete vitrification-based procedure comprises several steps: pretreatment, preconditioning, preculture, osmoprotection, dehydration, cooling, warming, dilution, and regrowth [100,101,102]. All these steps must be optimized, so that cells are able to vitrify, after dehydration, upon rapid cooling in LN without any damage resulting from freezing and/or the high toxicity of the vitrification solutions [101]. Several preculture media were investigated, prior to cryopreservation by vitrification, for shoot tips of *Anigozanthos viridis* ssp *terraspectans* Hopper [84], and somatic embryos of *Macropidia fuliginosa* (Hook.) Druce [86], plant species endemic to the southwest of Western Australia. The survival of the explants after rewarming was improved up to 84% and 90.6%, respectively, proving that the composition of the preculture medium has a significant impact on the regeneration of cryopreserved germplasm. Another vital step for an effective cryopreservation protocol by any vitrification-based procedure is the exposure to the vitrification solution. Shoot tips of *T. moroderi* [80] and *T. major* [82], were exposed to PVS2 between a time range from 0 to 120 min. The highest survival rates, 71.4% and 60%, respectively, were achieved after 60 min of exposure for both species. Pollinia of *L. macranta*, also subjected to several PVS2 exposure times, showed 56% germination after 10 min [75]. Overall, the vitrification technique is fast, because it reduces the time needed to dehydrate samples, and reaches high recovery levels. The drawbacks are the difficulty to handle simultaneously numerous and small explants and that it requires careful timing and cryoprotectant solutions that can be toxic to certain plants [56,99].

The encapsulation-dehydration technique consists of the encapsulation of plant material in calcium alginate beads which are partially dehydrated, before rapid exposure to LN [14,17,103]. The rewarming step is simplified because the beads containing the plant material, once properly dehydrated, form a stable structure which prevents devitrification and therefore the formation of ice crystals [56]. An example of an endemic species cryopreserved using this method is *C. rigualii*, that achieved a survival of 70%, after 4 h desiccation [93]. Despite the advantages, this technique has not been largely used for the cryopreservation of endemic plant species. The complete technique comprises several steps and is very time demanding [56], which may limit its application.

The encapsulation-vitrification technique is a combination of encapsulation-dehydration and vitrification procedures. Plant material is encapsulated in calcium alginate beads and then subjected to dehydration using vitrification solutions [100]. This method was compared with encapsulation-dehydration in the cryopreservation of *Hladnikia pastinacifolia* Rchb., an endemic species of Slovenia [62]. The best regrowth percentages were obtained with 85 min exposure to PVS2, for encapsulation-vitrification, and 150 min desiccation with silica gel for encapsulation-dehydration, 64% and 53%, respectively. The authors considered that both methods can be applied for the cryopreservation of *H. pastinacifolia*. The encapsulation-vitrification merges advantages of the conventional vitrification and encapsulation-dehydration techniques: fast procedure and easy to manipulate encapsulated plant material [100,101]. However only the previous publication mentioned was found using this method for the cryopreservation of endemic plant species. As demonstrated by the example above, different techniques can be effectively applied to the same plant species and obtain similar results [14,104]. Close regrowth percentages were also obtained in the cryopreservation of *T. major* using two different methods [82]. The techniques investigated were vitrification and encapsulation-dehydration with regrowth percentages of 60% and 67%, respectively.

#### 4.2.2. Droplet-Vitrification

One of the most successful cryopreservation techniques is the droplet-vitrification technique, which is a combination of conventional vitrification and the droplet-freezing technique developed by Kartha et al. [105,106]. In this procedure, plant material is placed in droplets of cryoprotectant solution, on small aluminum foil strips, before rapid immersion in LN [14,101]. The use of a minimum volume of cryoprotective solution allows ultra-rapid cooling and warming rates [56,101]. However, there is a contamination risk since the set droplet/plant material is directly exposed to LN [56]. As with the conventional vitrification technique, the various steps of the process must be optimized. There are some reports that describe the optimization of the several steps of the cryopreservation protocol for endemic plant species. For *Aster altaicus* var. *uchiyamae* Kitam, an endemic species to Korea, the conditions that gave the best regeneration results, 65.3%, were loading solution (17.5% glucose + 17.5% sucrose) at 0 °C for 60 min, followed by exposure to an altered PVS2 solution (33.3% glucose + 13.3% DMSO + 13.3% ethylene glycol + 20.1% sucrose) at 0 °C for another 60 min [87]. These authors also proved that preculture had no effect on the regeneration of *A. altaicus* cryopreserved shoot tips, but the composition of the recovery medium was relevant to improve shoot tips regrowth. A regeneration of 86% was achieved for *P. mileense* when shoot tips were precultured with 0.3 M sucrose for 24 h and then exposed to PVS2 for 90 min at 0 °C, before immersion in LN [79]. Regeneration of *P. mileense* shoot tips was not improved by cold acclimation. *Thymus lotocephalus* G. López & R. Morales, endemic to the south of Portugal, attained 67% recovery after subculture of *in vitro*-donor plants at 25 °C for four weeks, preculture of shoot tips for one day on MS medium containing 0.3 M sucrose and 60 min exposure to PVS2 [91]. *Cycladenia humilis* Benth. var. *jonesii* (Eastw.) S.L. Welsh & N.D. Atwood, endemic to Utah and Arizona, USA, was cryopreserved by droplet-vitrification and the success in the recovery of the shoot tips after exposure to LN was determined by the recovery medium composition [33]. Testing different exposure times to the vitrification solution is essential during the optimization of cryopreservation protocols by droplet-vitrification. Other steps of the procedure appear to have a minor impact on the regeneration of cryopreserved germplasm from endemic plant species using this technique.

#### 4.2.3. Cryo-Plate Methods

Some of the most recent and innovative cryopreservation procedures use cryo-plates: V cryo-plate and D cryo-plate methods. Yamamoto et al. [107] created an aluminum plate with small wells (cryo-plate) where explants can be placed. This was the basis for the development of a new cryogenic procedure based on vitrification dehydration, the V cryo-plate method [57,102]. The method is similar to the droplet-vitrification technique, but instead of an aluminum foil stripe, a cryo-plate is used. Before placing the plant material, droplets of calcium alginate are added to the cryo-plate wells to better adhere it to the plate. Attaching the explants to the cryo-plate enables an easier manipulation throughout the process of loading, exposure to the vitrification solution, immersion in LN, and thawing [20,102]. This method is highly advantageous because by attaching the explants to the cryo-plate, there is no need to move them directly, avoiding their damage and/or loss. The very rapid cooling and warming rates protect the explants from cryogenic injuries allowing high regrowth rates. Besides, the implementation and training for this technique is simple and fast [20,57,102,107]. The D cryo-plate method is a combination of V cryo-plate and encapsulation-dehydration. Explants placed in the cryo-plate wells are air-dehydrated in a laminar flow cabinet, instead of being exposed to a vitrification solution, before immersion in LN [57,102,108]. In addition to the advantages of the V cryo-plate method, D cryo-plate can be implemented with larger samples and avoids the toxicity of cryoprotectant solutions [102,108]. The specific construction requirements of the cryo-plates can be limitative to its use, especially at a large scale [20,94]. A stainless-steel mesh strip (cryo-mesh) was created by Funnekotter et al. to overcome this issue [94]. The cryo-mesh is fabricated using wire mesh strips which are simpler to obtain. Its structure enables a faster infiltration of the cryo-solutions and the use of plant material of different dimensions and shapes [94]. The cryo-mesh was tested in the cryopreservation of *A. viridis* shoot tips and compared to the droplet-vitrification technique. A regeneration of 83% was obtained after 30 min exposure to PVS2 using the cryo-mesh. There were no significant differences when compared to the droplet-vitrification technique, which resulted in 78% regeneration. The use of cryo-mesh greatly simplifies the cryopreservation process by reducing the handling of the plant material, in comparison with standard droplet-vitrification [20,94].

#### 4.2.4. Vacuum-Infiltration Vitrification (VIV)

The vacuum infiltration vitrification (VIV) technique [109] is another recent and innovative cryopreservation method, also based on the conventional vitrification technique. In this technique, vacuum is used to speed the infiltration of the cryoprotectant solution into plant material, therefore reducing the total incubation time required before vitrification, and allowing higher regrowth rates [109]. The vacuum allows a more uniform penetration of the cryoprotectant solutions because it increases the contact between the cryoprotectant solution and cell membranes by reducing intracellular air on the surface of plant material to be cryopreserved [20,102,109]. This technique was tested on shoot tips of several species endemic to southwest Western Australia, that already had cryopreservation protocols developed, such as *A. viridis* ssp *terraspectans* and *L. cinerea*, above-mentioned in this manuscript, and *Lomandra sonderi* (F.Muell.) Ewart. Overall, comparing to droplet-vitrification, the VIV technique significantly reduced optimal PVS2 incubation time for cryoprotection and improved the survival rates in the regeneration of cryopreserved shoot tips of all the species studied. In the case of *L. cinerea*, no regeneration was achieved after shoot tip cryopreservation with conventional droplet-vitrification [95]. This had also been proved earlier by Kaczmarczyk et al. [78], which replaced shoot tips for callus. However, with the VIV technique, up to 10% regeneration of *L. cinerea* shoot tips was obtained after 10 min exposure to PVS2 under vacuum conditions [95].

### 4.3. Studies to Evaluate Cryogenic Effects

The quality and survival of plant material after storage is extremely important for any conservation protocol to succeed. During the entire cryopreservation process, which includes different stages, from the *in vitro* culture until the regeneration of complete plants from the cryopreserved plant material, plant cells are subjected to a variety of stresses which may induce morphological and cytological variations and affect their genome, resulting in low regeneration after thawing [14,58,110]. This is particularly risky for endemic plant species because the number of populations and individuals is frequently limited in these species, and any additional loss of genetic diversity may be unmanageable. Therefore, learning and investigating how the different factors of the cryogenic procedure can interfere in the final result is fundamental for the development of effective cryopreservation protocols.

As previously described in the beginning of the current paper, preventing ice formation is pivotal for the success of any cryopreservation procedure and in recent years, new methods have been used to study its effects. There are a few techniques that allow to observe and measure ice formation in cells and tissues and therefore are important tools to assist in the development of cryopreservation protocols. Low temperature scanning electron microscopy is useful in the monitorization of biological material which can be used to observe intra- and extra-cellular ice in conditions similar to those of cryopreservation procedures [111]. Differential scanning calorimetry (DSC) is a thermophysical analysis method that can measure ice formation in plant material during cryopreservation [78,89]. The optimization of PVS2 exposure time of *L. sonderi* shoots tips, an endemic plant of southwest Western Australia, was assisted by thermal analysis using DSC to detect ice formation in their cells [89]. Other parameters, such as preconditioning of *in vitro*-donor plants and preculture medium composition and duration, were also assessed, but the thermal analysis proved that only PVS2 exposure time influenced the reduction of ice formation. Thermal analysis using DSC also confirmed that PVS2 treatment prior to cryopreservation prevents ice formation in shoot tips of *L. cinerea* [78].

Another factor that can cause damages to cells is oxidative stress, that results from the accumulation of reactive oxygen species, and can appear at any step of the cryopreservation procedure [20,110]. Oxidative stress can be determined, for instance, by measuring the activity of antioxidant enzymes. The antioxidant activity of various enzymes, exposed to different preconditioning conditions, was analyzed along the cryopreservation process of *L. sonderi* [112]. The activity of glutathione reductase decreased in the recovery stage, after cryopreservation, while the activity of glutathione peroxidase and catalase remained equal. In addition, superoxide dismutase presented a positive correlation between post-cryopreservation survival and antioxidant activity, but other enzymes, glutathione reductase, glutathione peroxidase and catalase, presented no correlation [112]. The antioxidant defense against oxidative stress was evaluated for recovered plants from cryopreserved shoot tips of *Hypericum rumeliacum* Boiss., an endemic species to the Balkans [85]. The results showed that high oxidative stress, provoked by the preculture treatment, decreased the enzymatic antioxidant defense of regenerated cryopreserved material after long culture periods. However, cryopreservation did not affect the capacity of *in vitro* cultured plantlets to produce phenolics and flavonoids [85].

The assessment of the genetic integrity of cryopreserved plant material is also important to understand the viability of the procedures applied. Several techniques can be employed to assess the genetic stability of cryopreserved plant material, such as phenotypic, cytological, biochemical and molecular [58]. The number of studies concerning plant germplasm integrity after cryopreservation storage is very limited for endemic plant species. Nevertheless, the studies performed so far demonstrated that there are no differences or very few variations between cryopreserved and non-cryopreserved plant material [62,81,85,91]. Random amplified polymorphic DNA (RAPD) is one of the most used molecular techniques for the assessment of genetic stability, including for endemic species. [62,91]. The genetic stability of *T. lotocephalus* cryopreserved shoot tips was assessed using RAPD markers [91]. Variations at a low frequency (0.06%) were observed, although these had no influence on the morphological characteristics of the plants recovered from the cryopreserved shoot tips. *T. moroderi* plants, derived from cryopreserved shoot tips, one month after acclimatization to *ex vitro* conditions were assessed for their genetic and phytochemical stability [113]. A variation of 0.34% was detected using RAPD markers and no morphological differences were detected between plants from cryopreserved and non-cryopreserved plant material. The phytochemical analysis was performed by GS-MS and the results demonstrated that the major components found were the same as those usually found in *T. moroderi* wild plants. The variations observed during the genetic stability studies might not be phenotypically perceptible because they only affect non-coding regions. On the other hand, RAPD is a molecular technique that screens a low fraction of the genome and some genetic changes are not detected [58].

## 5. Synopsis and Future Perspectives

By combining *in vitro* propagation with cryopreservation, a powerful strategy is created. The development and optimization of *in vitro* propagation protocols is fundamental to ensure the propagation of plants after the recovery of the stored plant material independently of the *in vitro* strategy followed. Plants raised *ex situ* can be then used to restore or reinforce wild depauperated populations and for other applications including breeding programs. Cryopreservation secures the plant material in cryobanks, in theory for an unlimited time, for further use in plant production for many applications including the re-introduction in the wild [56]. However, the publications available in international journals concerning the cryopreservation of endemic plant species is still limited, considering their global distribution and number. The overall conclusion is that much more can be done in the future to benefit from cryopreservation in the conservation of these unique plant species. Several causes can be pointed out to why the number of scientific publications is not high in this matter:

- *Unpublished data*: there could be a reasonable number of protocols that were developed at cryobanks or botanic gardens for specific endemic species that are been used but have not been published;
- *Low funding*: the investment in the conservation of wild plants is not retrieved as the investment on crop, medicinal and ornamental species. Their economic value makes these last species more engaging for research and therefore more accessible to funds, not only from funding agencies at an academic level but also from private investors, leaving few resources for the conservation of endemic and rare species. In addition, there are considerably more funding and resources for the conservation of animals rather than plants [114,115];
- *Scarce plant material*: endemic plant species frequently occur in small populations in the wild, occasionally with difficult access, and thus the plant material available is very limited. In addition, a considerable number of endemic species are legally protected and, therefore, subjected to restrictions for their use and collection [115];
- *No standard protocols*: protocol development and optimization is the most labor-intensive phase of cryopreservation [56]. In the case of endemic species, their unique nature and often unknown biology and physiology makes it even harder to determine the best conditions for cryopreservation and almost impossible to establish standard protocols.
- *Cryobank facilities*: although to develop a cryopreservation protocol, with the vitrification-based techniques, only a standard tissue culture lab is necessary, to effectively store germplasm under cryogenic conditions more complex and costly facilities are required. Not always laboratories that develop the cryopreservation protocols are prepared to maintain germplasm for long periods of time. The scale-up of the germplasm and cryopreservation procedures requires the establishment of specific measures for their management [14]. Priority is given once again to species economically more desirable.
- *High number*: presently it is not possible to extend conservation efforts to all endemic species. There are just too many and too less resources to cover all species.

The limitations in the preservation of endemic plant species are variable because of their high diversity, geographic range, and/or accessibility. Research on this matter is urgent and the potential for future scientific studies on the cryopreservation of endemic plant species is enormous. There are considerably more studies on *in vitro* propagation than cryopreservation for the conservation of endemic plant species. Despite all the advantages of cryopreservation, this technique is still more costly and difficult to implement when compared to *in vitro* plant propagation. Nonetheless, there have been efforts made in different parts of the world to create cryopreservation protocols for these unique species. The scientific publications found (information obtained using a combination of the keywords “cryopreservation+endemic+plant+species” via Science Direct, Google Scholar, and standard Google search, in the English language) related to the development of cryopreservation protocols for endemic plant species were less than ten until 2009. Between 2010–2014, there was a substantial increase, approximately twenty publications; however, that number decreased after 2015 until the present and most publications are related to improvements in protocols and not the report of new protocols for other species. It is interesting to notice that many of the publications regarding the cryopreservation of endemic plants belong to species that occur in Biodiversity Hotspots. These are identified areas all over the planet with high concentration of endemic species and high habitat loss. There are 35 Biodiversity Hotspots that cover only 17.3% of the land surface of the Earth but maintain 77% of all endemic plant species [12]. Cryopreservation protocols were established, for instance, for species from the Biodiversity Hotspots Mediterranean basin (*T. lotocephalus* [91], *T. moroderi* [80,81], *T. major* [82], *P. algarbiensis* [70], *C. rigualii* [93], among others); Southwest Australia (*L. sonderi* [89,95], *L. cinerea* [78,95], *M. fuliginosa* [86] and *A. viridis* spp *terraspectans* [83,84,95]); and Western Ghats and Sri Lanka (*L. macranta* [75] and *N. nimmoniana* [74]).

The resources available are not enough to preserve all biodiversity and a recognized conservation strategy is to direct efforts and prioritize the preservation of species in areas rich in endemic species, such as the Biodiversity Hotspots [13]. However, within these areas, the number of plant species is still very high, considering the existing means. Since not all endemic species are rare, the identification of the species more vulnerable, i.e., with restricted ranges and low-density populations, is therefore very important in the selection of biodiversity to be preserved [7]. Once identified, the conservation planning for these species should encompass combined conservation strategies, including seed banking and *in vitro* techniques, and the setting of conservation objectives [4,7].

Other essential aspects to establish an efficient conservation program are the understanding of the natural populations’ structure and the assessment of the species’ genetic diversity, particularly when it includes *ex situ* conservation measures, such as cryopreservation [7,19,116]. Storage methods should comprise extensive collections with representative genetic diversity. Genetic variation is the basis for evolution and the plant material to be preserved *ex situ* should be demonstrative of the wild population diversity it represents, so that in the future, if need be, the material can be used for restoration of natural populations [19]. Classic molecular markers, such as RAPD, RFLP (restriction fragment length polymorphism), AFLP (amplified fragment length polymorphism), and ISSR (inter simple sequence repeats) were very useful in the study of genetic diversity and in the identification of species [19,117], although nowadays there are more updated methods. The number of publications reporting the genetic diversity of wild populations from endemic plant species is considerably higher than the publications regarding cryopreservation. As for the species mentioned in this review, a few were assessed for the genetic diversity of their wild populations: *C. humilis* [118], *A. microphyllum* [119], *Oxytropis chankaensis* Jurtz. [120], *H. pastinacifolia* [121], *T. major* [122], *P. mileense* [123], *P. algarbiensis* [116,124], *Pitcairnia encholirioides* L.B.Sm. [65], *N. nimmoniana* [125] and *Encholirium spectabile* Martius ex Schultes f. [126]. Recommendations for their conservation were given according to the results and the characteristics of the populations from each species. Though in some cases the genetic diversity studies were performed after the development of *in vitro* conservation techniques, the information obtained about the structure and genetic diversity of those populations will be very useful for further collections and storage of plant germplasm from these species. The next-generation sequencing methods, which are faster and less expensive than the classical ones, can facilitate the connection between molecular markers and conservation management. These new molecular techniques can identify thousands of markers in a single step and evaluate metabolisms even for unknown genomes, which is the case for most endemic plant species [2,117]. Examples of these methods are DNA microarray and their derived methods, diversity array technology (DArT) and subtracted diversity array (SDA), which are more appropriate for species with no previous knowledge of the genome [117].

As for cryopreservation itself, a deeper knowledge about the overall extent of the cryopreservation process could assist in a faster development of new protocols or even in the creation of easy-to-use procedures. Several factors and their interactions influence the success of any cryopreservation protocol. Understanding these factors, such as plant physiology and stress tolerance, will contribute to a faster and easier optimization of new cryopreservation procedures [109,110]. The advances in metabolomic, genomic, transcriptomic, and proteomic technologies are very promising for the acquisition of more detailed information about the physiological, biochemical, molecular, and ultrastructural changes that plant material undergoes during cryogenic storage. These techniques can help finding where (in the plant tissues) and/or what (cryoprotectant, ice crystals, etc.) exactly is damaging the cells, increase the knowledge on cryoinjury, and, consequently, facilitate the development of cryopreservation protocols by solving specific problems [102,110]. This information coupled with data from protocols already developed for closely related species or species from similar habitats can be very beneficial in the successful regeneration of cryopreserved plant germplasm [102]. Ultimately, time and resources spent on the development of cryopreservation protocols for endemic plant species can be significantly reduced.

The innovative cryopreservation techniques recently developed, cryo-plate methods and VIV, are important improvements for the cryopreservation of plant gemplasm. The application of these methods may ease and accelerate the development of new protocols due to their novel approaches to the whole cryopreservation process. Cryo-plate methods are simple and easy to use and could be applied for large scale storage in cryobanks [107]. As for VIV, this method reduces the exposure time to cryoprotectants, thus reducing their toxicity, and enables high regrowth percentages [109]. These novel methods should be tested for the cryopreservation of germplasm of more endemic plant species or even in the improvement of protocols already established.

Overall, a deeper knowledge on the cryopreservation process allied to new technologies can greatly aid and facilitate the development of simpler and standardized procedures that can ultimately be used by a wider range of institutions and laboratories and therefore augment the efforts on the preservation of endemic plant species worldwide.

Concerns about the environment, climate changes, earth’s genetic heritage, mass extinction, and weather alterations are increasing worldwide. The general public is now more aware of the importance of endemic plant species, their unique genetic material, and the need to preserve them. Hopefully, this increasing interest will lead to the expansion of the resources available to support the preservation of endemic plant species. Besides, the new technologies available can greatly facilitate and haste not only the development of new cryopreservation protocols but also in the establishment of a bridge between *in situ* and *ex situ* conservation strategies. There is an urgent need to expand conservation research and particularly transfer the academic knowledge acquired to the actual implementation of conservation strategies in practice. The cryopreservation of endemic plant species is a challenge and there is a considerable amount of work that needs to be done in a near future to complement the preservation of these unique plant species and prevent their extinction in the wild. Furthermore, it is important to promote studies that can confirm the economic value of many unstudied and unexplored endemic plant species, namely their potential for the extraction of valuable bioactive compounds, for biofuels production, for bioremediation, among others. This may increase the interest in these plants and encourage the implementation of conservation strategies. As above-mentioned in this manuscript the *in vitro* techniques allow the mass propagation of plants and therefore the sustainable use of plant biomass for many applications.

## Figures and Tables

**Table 1:** Selected scientific publications on micropropagation of endemic plant species, including the name of the species, the region of distribution, and the explant type.

Species	Region	Explant	Reference
*Aechmea ramosa* Mart. ex Schult. f.	Atlantic Forest (Brazil)	Leaf	[26]
*Asparagus macrorrhizus* (Pedrol, Regalado & López-Encina)	“Mar Menor” lagoon, Murcia (Spain)	Rhizome buds	[27]
*Brachystelma glabrum* Hook.f.	Eastern Ghats (India)	Shoot tips and nodal explants	[28]
*Calophyllum apetalum* Willd.	Western Ghats of southern India	Shoot tips and nodal explants	[29]
*Ceropegia noorjahaniae* Ans.	Western Ghats (India)	Shoot segments (node explant)	[30]
*Coelogyne nervosa* A. Rich.	Western Ghats (South India)	Seedlings	[31]
*Cryptanthus sinuosus* (L.B. Smith)	Rio de Janeiro State (Brazil)	Stem–root axes with intact shoot apices and approximately 6–10 nodes	[32]
*Cycladenia humilis* Benth. var. *jonesii* (Eastw.) S.L. Welsh &N.D. Atwood	Canyonlands region of Utah and Arizona (Western U.S.A.)	Seedlings and nodal segments	[33]
*Dianthus giganteus* D’Urv subsp. *banaticus* (Heuff.) Tutin	Southwest Carpathians (Romenia)	Nodal explants	[34]
*Dianthus pinifolius* Sibth. et Sm.	South-eastern Balkans	Seedlings	[35]
*Eryngium viviparum* Gay	Southwest Europe (France, Spain and Portugal)	Seedlings	[24]
*Henckelia incana* (Vahl) Spreng.	Peninsular hills of India	Leaves	[36]
*Hladnikia pastinacifolia* Rchb	Plateau of Trnovski Gozd, Alps (Slovenia)	Seedlings and shoots	[37]
*Hypericum richeri* ssp. *transsilvanicum* (Čelak) Ciocârlan	Southeast Carpathians (Romania)	Seedlings (nodes, leaves, internodal stems and and root segments)	[38]
*Juniperus navicularis* Gand	Southwest Portugal	Shoot tips and nodal segments	[39]
*Juniperus thurifera* L.	Aure‘s Mountains (Northeastern Algeria)	Shoots	[40]
*Magnolia dealbata* Zucc.	Mexico (south–central)	Zygotic embryos	[41]
*Laelia anceps* Lindl	Mexico	Seedlings	[42]
*Leucocroton havanensis* Borhidi	Cuba	Seedlings	[43]
*Picconia azorica* (Tutin) Knobl.	Azores (Portugal)	Nodal segments	[44]
*Plantago algarbiensis* Samp.	Algarve Region (Portugal)	Seedlings	[45]
*Plantago almogravensis* Franco	Portuguese Southwest coast	Seedlings	[45]
*Quercus euboica* Pap.	Northeastern Euboia island (middle eastern Greece)	Seedlings	[46]
*Quercus lusitanica* Lam.	Iberian Peninsula and North Africa	Shoots	[47]
*Reseda pentagyna* Abdallah & A.G. Miller	Saudi Arabia	Axillary and apical buds	[48]
*Salvia valentina* Vahl and *Salvia blancoana* Webb & Heldr subsp. *mariolensis* Figuerola	Valencia Community (Spain)	Apical and nodal segments	[49]
*Thymus lotocephalus* G. López & R. Morales	Algarve Region (Portugal)	Seedlings	[50]
*Tuberaria major* (Willk.) P. Silva and Rozeira	Algarve Region (Portugal)	Seedlings	[51]
*Zelkova sicula* Di Pasquale, Garfì and Quézel	South-eastern Sicily (Italy)	Shoot segments	[52]

**Table 2:** Scientific publications on the cryopreservation of seeds of endemic plant species (after 2010). Data refers to maximum germination rate obtained after cryopreservation. The IUCN Status 2020 is also included (NE—Not Evaluated; DD—Data Deficient; LC—Least Concern; NT—Near Threatened; VU—Vulnerable; EN—Endangered; CR—Critically Endangered).

Species	Germination Rate (%)	Reference	IUCN
*Encholirium spectabile* Martius ex Schultes f.	97	[67]	LC
*Ermania parryoides* Cham. Ex Botsch.	70	[66]	-
*Ferula iliensis* Krasn. ex Korov	90	[68]	-
*Hedysarum austrokurilense* (N.S. Pavlova) N.S. Pavlova	85	[66]	-
*Hedysarum sachalinense* B. Fedtch. (Fabaceae)	75	[66]	-
*Melocactus conoideus* Buining and Brederoo	10	[69]	CR
*Micranthocereus flaviflorus* subsp. *densiflorus* (Buining and Brederoo) P. J. Braun and Esteves	59	[69]	NT
*Micranthocereus polyanthus* subsp. *alvinii* M. Machado and Hofacker	23	[69]	EN
*Myosotis sachalinensis* M. Pop.	30	[66]	-
*Oxytropis chankaensis* Jurtz.	75	[66]	-
*Oxytropis kamtschatica* Hult.	90	[66]	-
*Oxytropis retusa* Matsum.	40	[66]	-
*Pitcairnia encholirioides* L.B.Sm.	85	[65]	-
*Plantago algarbiensis* Samp.	100	[70]	EN
*Saxifraga purpurascens* Kom.	20	[66]	-
*Stellaria eschscholziana* Fenzl	35	[66]	-
*Tanacetum ulutavicum* Tzvel.	72	[71]	-
*Thymus lotocephalus* G. López & R. Morales	90	[63]	NT
*Tuberaria major* (Willk.) P. Silva and Rozeira	65	[64]	EN
*Vaccinium vulcanorum* Kom.	90	[66]	-
*Vicia subrotunda* (Maxim.) Czefr.	50	[66]	-

**Table 3:** Scientific publications on cryopreservation of endemic plant species, by method (D-V—Droplet-vitrification; E-D—Encapsulation-dehydration; E-V—Encapsulation-vitrification; Direct LN—Direct immersion in liquid nitrogen; Vit—Vitrification) and explant type. Data refers to maximum survival (S)/regrowth (R)/germination (G) rate obtained after cryopreservation. The IUCN Status 2020 is also included (NE—Not Evaluated; DD—Data Deficient; LC—Least Concern; NT—Near Threatened; VU—Vulnerable; EN—Endangered; CR—Critically Endangered).

Method(s)	Species	Region	Explant	Rate (%)	Reference	IUCN
Vit	*Anigozanthos viridis* ssp *terraspectans* Hopper	Western Australia	Shoot tips	89 (S)	[83,84]	-
	*Hypericum rumeliacum* Boiss.	Balkan Peninsula	Shoot tips	2 (S)	[85]	-
	*Luisia macrantha* Blatt. & McCann	Western Ghats (India)	Pollinia	56 (G)	[75]	-
	*Macropidia fuliginosa* (Hook.) Druce	Western Australia	Shoot tips Somatic embryos	84 (S) 91 (S)	[86]	-
	*Thymus moroderi* Pau ex Martínez	South-eastern Spain	Shoot tips	70 (S)	[81]	-
	*Tuberaria major* (Willk.) P. Silva and Rozeira	Algarve Region (Portugal)	Shoo tips	60 (R)	[82]	EN
D-V	*Aster altaicus* var. *uchiyamae* Kitam	Korea	Shoot tips	65 (R)	[87]	-
	*Cycladenia humilis* Benth. var. *jonesii* (Eastw.) S.L. Welsh & N.D. Atwood	Canyonlands region of Utah and Arizona (USA)	Shoot tips	54 (R)	[33]	-
	*Dianthus giganteus* D’Urv subsp. *banaticus* (Heuff.) Tutin	Southwest Carpathians (Romenia)	Shoot tips	43 (R)	[34]	-
	*Hypericum richeri* ssp. *transsilvanicum* (Čelak) Ciocârlan	Southeast Carpathians, Transilvania (Romania)	Axillary buds	68 (S)	[38]	-
	*Lithodora rosmarinifolia* (Ten.) I. M. Johnst.	Sicily (Italy)	Nodal segments	33 (R)	[88]	-
	*Lomandra sonderi* (F.Muell.) Ewart	Western Australia	Shoot tips	32 (S)	[89]	-
	*Loxocarya cinerea* R. Br.	Western Australia	Callus	90 (S)	[78]	-
	*Paraisometrum mileense* W. T. Wang	Yunnan, China	Shoot tips	86 (R)	[79]	-
	*Plantago algarbiensis* Samp.	Algarve Region (Portugal)	Nodal segments	60 (R)	[70]	EN
	*Thymus cariensis* Hub-Mor. & Jalas	Turkey	Shoot tips	25 (R)	[90]	-
	*Thymus lotocephalus* G. López & R. Morales	Algarve Region (Portugal)	Shoot tips	67 (R)	[91]	NT
	*Thymus moroderi* Pau ex Martínez	South-eastern Spain	Shoot tips	79 (S)	[80]	-
E-D	*Antirrhinum microphyllum* Rothm.	Spain	Nodal segments	70 (S)	[92]	-
	*Hladnikia pastinacifolia* Rchb	Plateau of Trnovski Gozd, Alps (Slovenia)	Shoot tips	53 (R)	[62]	DD
	*Centaurium rigualii* Esteve	Iberian Peninsula	Nodal segments	70 (S)	[93]	-
	*Plantago algarbiensis* Samp.	Algarve Region (Portugal)	Nodal segments	63 (R)	[70]	EN
	*Pteris adscensionis* Swartz	Ascension Island	Gametophytes	48 (S)	[21]	CR
	*Thymus moroderi* Pau ex Martínez	South-eastern Spain	Shoot tips	50 (S)	[81]	-
	*Thymus lotocephalus* G. López & R. Morales	Algarve Region (Portugal)	Shoot tips	44 (R)	[91]	NT
	*Tuberaria major* (Willk.) P. Silva and Rozeira	Algarve Region (Portugal)	Shoot tips	67 (R)	[82]	EN
E-V	*Hladnikia pastinacifolia* Rchb	Plateau of Trnovski Gozd, Alps (Slovenia)	Shoot tips	64 (R)	[62]	DD
Cryo-mesh	*Anigozanthos viridis* Endl.	Western Australia	Shoot tips	83 (R)	[94]	-
VIV	*Anigozanthos viridis* ssp. *terraspectans* Hopper	Western Australia	Shoo tips	31 (R)	[95]	-
	*Lomandra sonderi* (F.Muell.) Ewart	Western Australia	Shoo tips	42 (R)	[95]	-
	*Loxocarya cinerea* R. Br.	Western Australia	Shoo tips	10 (R)	[95]	-
Desiccation	*Nothapodytes nimmoniana* (Graham) Melbbery	Western Ghats (India)	Embryonic axes	60 (G)	[74]	-
Direct LN	*Pleopeltis lepidopteris* Langsd. & Fisch.	South Brazil	Spores	89 (G)	[96]	-
